# Age-related change in children’s physical activity and sedentary time: The International Children’s Accelerometry Database (ICAD)

**DOI:** 10.1371/journal.pone.0327394

**Published:** 2025-09-10

**Authors:** Andrew J. Atkin, Lauren B. Sherar, Ulf Ekelund, Bjorge H. Hansen, Lars Bo Andersen, Sigmund Anderssen, Susi Kriemler, Soyang Kwon, Peter L. Kristensen, Niels Wedderopp, Kate Northstone, Russell Jago, Russell Pate, Jardena J. Puder, Jo Salmon, Luis B. Sardinha, Esther M.F. van Sluijs, on behalf of the International Children’s Accelerometry Database (ICAD) collaborators

**Affiliations:** 1 School of Health Sciences, Faculty of Medicine and Health Sciences, University of East Anglia, Norwich, United Kingdom; 2 MRC Epidemiology Unit, University of Cambridge School of Clinical Medicine, Institute of Metabolic Science, Cambridge Biomedical Campus, Cambridge, United Kingdom; 3 School of Sport, Exercise and Health Sciences, Loughborough University, Loughborough, United Kingdom; 4 Department of Sport Medicine, Norwegian School of Sport Science, Oslo, Norway; 5 Department of Chronic Diseases, Norwegian Institute of Public Health, Oslo, Norway; 6 Department of Sport Science and Physical Education, University of Agder, Kristiansand, Norway; 7 Faculty of Teacher Education and Sport, Western Norway University of Applied Sciences, Sogndal, Norway; 8 Epidemiology, Biostatistics and Prevention Institute, University of Zürich, Zürich, Switzerland; 9 Department of Emergency Medicine, Buehler Center for Health Economics and Policy, Northwestern University, Chicago, United States of America; 10 Department of Sports Science and Clinical Biomechanics, University of Southern Denmark, Odense, Denmark; 11 Population Health Sciences, Bristol Medical School, University of Bristol, Bristol, United Kingdom; 12 Department of Exercise Science, University of South Carolina, Columbia, United States of America; 13 Obstetric Service, Department Woman-Mother-Child, Lausanne University Hospital, Lausanne, Switzerland; 14 Institute for Physical Activity and Nutrition Research, Deakin University, Melbourne, Australia; 15 Exercise and Health Laboratory, CIPER, Faculdade de Motricidade Humana, Universidade de Lisboa, Lisbon, Portugal; University of Tartu, ESTONIA

## Abstract

**Background:**

Many young people fail to achieve the minimum recommended amount of physical activity to benefit their health. Understanding the nature of age-related changes in behaviour and how this varies for population sub-groups is informative for intervention design. The aim of this study was to describe age-related changes in physical activity and sedentary time and examine variability in patterns of change across demographic sub-groups.

**Methods:**

Data are from 13 studies in the International Children’s Accelerometry Database (ICAD), all of which provided ≥2 waves of waist-worn accelerometer data. Annual change in sedentary time, light intensity physical activity (LPA) and moderate to vigorous intensity physical activity (MVPA) were estimated using three level mixed effects linear regression models, using participant age as the exposure variable. In interaction models, we examined whether changes in behaviour differed by day of the week (weekday/ weekend), age at baseline assessment (<10/ ≥ 10 years), sex, weight category, maternal education and ethnicity.

**Results:**

In total, 6567 participants provided two or more waves of valid accelerometer data (44.5% boys, mean (SD) baseline age 10.6 (2.1) years (range 4.1–15.8 years)). Across the week and for all studied sub-groups, sedentary time increased by approximately 25 minutes/day/year of age, LPA decreased by approximately 22 minutes/day/year of age and MVPA decreased by approximately 3 minutes/day/year of age. The annual increase in sedentary time was greater in girls compared to boys (β (95% confidence interval) change (min) for each additional year of age: girls, 25.9 (25.4, 26.4); boys, 23.6 (23.0, 24.2)) and in adolescents compared to children (adolescents, 27.0 (26.5, 27.6); children, (23.5 (22.9, 24.2)). The annual decrease in MVPA was greater in boys compared to girls (boys, −2.7 (−3.0, −2.5); girls, −2.2 (−2.4, −2.0)) and at the weekend compared to during the week (weekend, −3.0 (−3.3, −2.8); weekday −2.3 (−2.5, −2.1)).

**Conclusion:**

Accelerometer assessed sedentary time increased whilst LPA and MVPA decreased during childhood and adolescence. This overall pattern was observed across the week and in all studied sub-groups, but small differences in the magnitude of changes can be used to guide the timing and targeting of behaviour change interventions, such as designing physical activity interventions which focus on weekends where a child’s time is less structured.

## Introduction

The physical and mental health benefits of physical activity during childhood include favourable associations with aerobic and muscular fitness, cardiometabolic markers, depressive symptomology and cognitive function [1]. Accordingly, the World Health Organisation (WHO) recommends that young people aged 5–17 years, including those living with a disability, accumulate an average of 60 minutes of moderate to vigorous intensity physical activity daily and limit time spent sedentary [2]. Surveillance data, however, indicate that a substantial proportion of children and adolescents do not attain the minimum recommended levels of physical activity [3–5]. Against this backdrop, and the high prevalence of physical inactivity that persists into adulthood [6], the WHO Global Action Plan on Physical Activity targets a 15% relative reduction in physical inactivity globally by 2030 [7]. Achieving this goal will require high-quality evidence on patterns of activity during childhood, changes in behaviour with age and population sub-groups that may benefit from targeted intervention. Such evidence will help to inform the development of local- and national-level policies to promote physical activity and the design of tailored behaviour change interventions.

There is existing evidence of an age-related decline in human physical activity [8–10], corroborated by evidence of a similar phenomenon in non-human species [11]. However, previous studies on this topic using self- or proxy-reported measures of physical activity are susceptible to various forms of bias [12,13]. Device-based physical activity measurement has enabled researchers to more accurately describe the timing, magnitude and variability of physical activity change with age, but important gaps in the literature remain. Previous studies, for example, have examined sex differences in the age-related change in activity, but little evidence exists on how this may vary by socio-economic position or other demographic factors [9,14–16]. Further evidence of how activity patterns vary across social and demographic subgroups enables more precise development and targeting of intervention programmes, as does knowledge of whether behaviour patterns change differentially on weekdays versus the weekend [17]. In addition, most of the existing evidence is derived from single-country studies, with participants recruited from a relatively small geographic region, potentially limiting their generalisability [16,18]. Synthesis of previous research is hindered by between-study differences in data collection protocols and processing. The International Children’s Accelerometry Database (ICAD) is uniquely placed to address many of these limitations, it being the largest existing pooled repository of waist-worn accelerometry in young people, providing data from a number of geographically dispersed studies processed using a common protocol. The aims of this study were to (1) describe age-related changes in accelerometer-assessed physical activity and sedentary time and (2) examine whether changes vary across socio-demographic subgroups and day of the week (weekday/weekend).

## Methods

The ICAD is a repository of physical activity, demographic, behavioural, anthropometric and metabolic data collected in young people (<18 years) from 23 studies conducted in Europe, North and South America, and Australia. All studies used an Actigraph (Pensacola, FL) accelerometer, attached at the hip, to assess physical activity intensity and sedentary time. Data were collected between 2001−13 and pooled in 2016−17. All studies included in ICAD received ethical approval for their data collection and sharing of data and obtained written parental consent and child assent prior to data collection (further details available in study-specific publications). Standalone ethical approval for ICAD-related data pooling and harmonisation was not required. The original methods and subsequent update of ICAD have been described in detail previously [19,20]. The current analyses uses data from the second release of the database (ICAD2).

### Sample

ICAD studies that included two or more waves of accelerometer assessment were eligible for inclusion in the current study: Avon Longitudinal Study of Parents and Children (ALSPAC, UK) [21–23]; Ballabeina (Switzerland); Children Living in Active Neighbourhoods (CLAN, Australia); Copenhagen School Child Intervention Study (CoSCIS, Denmark); European Youth Heart Study Denmark (EYHS Denmark, Denmark); European Youth Heart Study Norway (EYHS Norway, Norway); European Youth Heart Study Portugal (EYHS Portugal, Portugal); Healthy Eating and Play Study (HEAPS, Australia); Iowa Bone Development Study (IBDS, USA); Kinder-Sportstudie (KISS, Switzerland); Personal and Environmental Associations with Children’s Health (PEACH, UK); Sport, Physical activity and Eating behaviour: Environmental Determinants in Young people (SPEEDY, UK); Trial of Activity for Adolescent Girls (Project TAAG, USA). For intervention studies (Ballabeina, CoSCIS, KISS, TAAG), only participants in the control groups were included in the analysis. Two waves of accelerometer data were available from Ballabeina, EYHS Denmark, EYHS Norway, EYHS Portugal, HEAPS, KISS and TAAG. Three waves of accelerometer data were available from ALSPAC, CLAN, CoSCIS, PEACH and SPEEDY. Participants in the IBDS completed up to six waves of accelerometer measurement. For consistency with other studies included here, only the first three waves of measurement for each participant were included in this analysis.

### Accelerometer data

Following a standardised protocol (available from http://www.mrc-epid.cam.ac.uk/research/studies/icad/), accelerometer files were reprocessed using Kinesoft software (V3.30, Loughborough UK). Raw Actigraph data files were reintegrated to 60-second epoch files to facilitate pooling. Periods of ≥60 minutes of consecutive zeros (with a tolerance of two minutes of non-zero interruptions) were classified as non-wear time and excluded, as was the period 11 pm to 6 am to minimise possible misclassification of overnight sleep as sedentary time. A valid day was defined as ≥600 minutes of valid wear time. A valid file was defined as ≥2 weekdays and ≥1 weekend day of valid accelerometer data. Only participants providing data for two or more waves of assessment were included in the analyses. Duration of sedentary time (≤100 counts per minute (CPM)), light (LPA, 101- < 2295 CPM) and moderate to vigorous intensity physical activity (MVPA, ≥ 2295 CPM) were estimated using previously validated count thresholds [24]. To account for differences in accelerometer wear time between waves of assessment, the duration of time in each activity intensity was calculated as the proportion of wear time in each intensity multiplied by the mean daily wear time across all waves of measurement. For example: weekday MVPA (wave 1) = (Mean daily MVPA (wave 1)/ Mean daily wear time (wave 1)) X Mean daily wear time (all waves). This standardisation was applied separately for all valid days of measurement, weekdays and weekend days.

### Exposure and moderator variables

The exposure variable was participant decimal age in years at each wave of measurement, calculated preferentially as elapsed time from date of birth to first day of accelerometer assessment. This was achieved for >90% of included data points. In the event of missing date of birth or date of measurement, we used an age variable provided by the original investigators or used date of completion for another study component (e.g., date of questionnaire completion or collection of anthropometric data) as a proxy for date of accelerometer assessment. A binary variable (baseline age < / ≥ 10 years) was derived to explore whether age-related change in activity intensity differed in children versus adolescents [25].

Participant sex was self or parent reported. Measured height (in metres) and weight (in kilograms) was used to ascertain participants’ age- and sex-specific BMI z-scores; weight status was categorized as underweight, normal weight or overweight/obese (implemented using ‘zanthro’ and ‘zbmicat’ in STATA, [26]). Maternal education, used as an indicator of family socioeconomic position (SEP), was classified as up to and including completion of compulsory education (low-mid SEP) or post-compulsory education/vocational training (high SEP). In all studies, maternal education was self- or partner-proxy reported, except for Project TAAG where it was child-reported. We used maternal education as reported at baseline for all studies, except Ballabeina where it was measured at the second wave of assessment (approximately 1 year after baseline) only. Participant ethnicity was categorised as white or non-white, due to limited heterogeneity in most of the included studies. Information on ethnicity was obtained by parental report (ALSPAC, EYHS Denmark, EYHS Norway, EYHS Portugal, IBDS, SPEEDY), child-report (Project TAAG), or researcher assessment (PEACH). Season of physical activity measurement was derived according to month of data collection and categorised as spring, summer, autumn and winter. Full details on the derivation of harmonised variables is available from the ICAD website (https://www.mrc-epid.cam.ac.uk/research/studies/icad/data-harmonisation/).

### Statistical analyses

Statistical analyses were conducted in STATA (14.2; StataCorp, College Station, Texas, USA). Descriptive statistics are presented as mean (standard deviation (SD)) or median (inter-quartile range (IQR)) for continuous variables and proportions for binary variables respectively. In preliminary analyses, absolute change (min/day) in each intensity category was calculated as follow-up minus baseline; for participants with three waves of valid accelerometer data we treated the third wave of data as follow-up for this calculation. Relative change (% baseline value) was calculated as (absolute change/ baseline)*100. Demographic and anthropometric characteristics of those included and excluded from the analysis were compared using chi-squared tests and independent samples t-tests as appropriate.

To examine age-related change in activity, sex- and season-adjusted, three level mixed effects linear regression models were used to estimate the annual change in each of the outcomes (sedentary time, LPA, MVPA), using all waves of data available for each participant. Levels pertained to time-points (level one), nested within participants (level two), nested within study (level three). The exposure variable was age (years) at each time point. The β coefficient for age indicates the per-year of age change in activity. Separate models were constructed for activity accumulated across all days of the week, weekdays and weekend days. Using data from all days of assessment, subsequent models were stratified by sex (male/ female), age at baseline (<10/ ≥ 10 years), body mass index category (underweight/ normal weight/ overweight and obese), maternal education (low-mid/ high) and ethnicity (white/ non-white). Lastly, using interaction terms in regression models, we tested for effect modification in the age effect by day of the week, sex, age at baseline, body mass index category (dichotomised as underweight and normal weight/ overweight and obese), maternal education and ethnicity.

## Results

**Fig 1:**
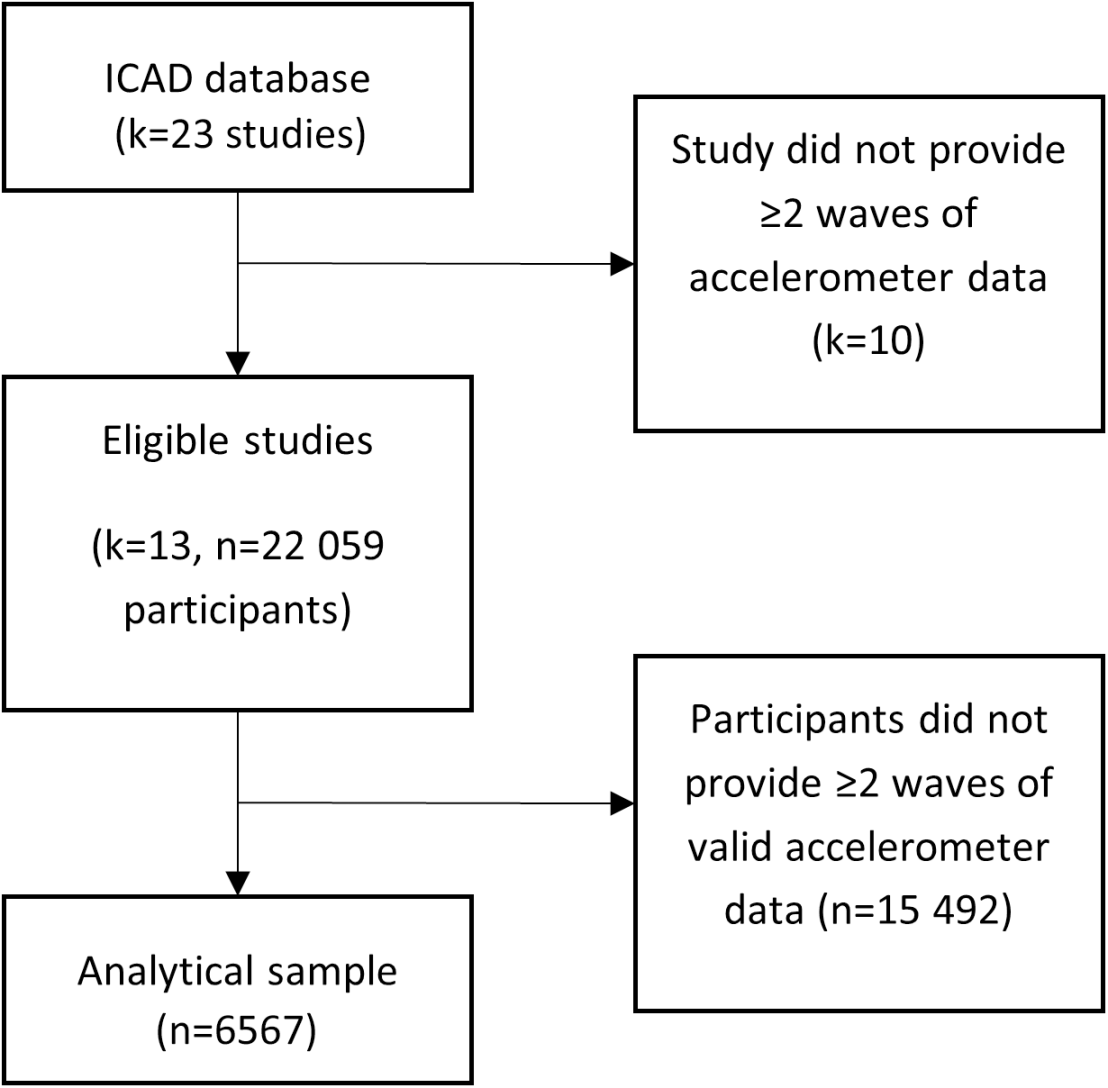
Flow chart for selection of the analytical sample.

From the 13 included studies, 22 059 participants were eligible for inclusion in the analyses, of which 6567 (29.8% of eligible) provided two or more waves of valid accelerometer data. A flow chart indicating the selection of the analytical sample is provided in Fig 1. Participant characteristics at baseline and elapsed time (years) between baseline and follow-up assessments are provided in Table 1. Compared to those excluded from the analysis, the analytical sample was younger (mean(SD) age at baseline: 10.6 (2.1) vs 11.6 (2.4) years, p < 0.01), had a lower BMI z-score (mean(SD) BMI: 0.34 (1.1) vs 0.51 (1.2), p < 0.01), and was more likely to be male (44.5% vs 36.8%, p < 0.01), of White ethnicity (89.2% vs 85.3%, p < 0.01) and have a higher educated mother (70.3% vs 56.1%, p < 0.01).

**Table 1:** Demographic characteristics of participants at baseline and follow-up duration (n = 6567)*.

Variable	Mean (SD) or %	
Sex (% male)	44.5	
Age (years)	10.6	(2.1)
BMI z-score	0.3	(1.1)
Ethnicity (% white)	89.2	
Maternal education (% low/medium)	26.9	
Duration of follow-up (years)**		
T_1_	2.5	(1.4)
T_2_	4.3	(1.1)

*Sample size denotes number of participants providing valid accelerometer data for at least 2 waves of assessment.

**Follow-up duration calculated as elapsed time in years from date of first day of accelerometer assessment at baseline (T_0_) to corresponding day at each subsequent wave of assessment.

SD, standard deviation; BMI, body mass index; SEP, socio-economic position.

Baseline, follow-up and change in daily time spent sedentary and in LPA and MVPA is presented in Table 2. At baseline, participants accumulated approximately 355 minutes (5.9 hours) of sedentary time, 388 minutes (6.5 hours) of LPA, and 56 minutes of MVPA per day. Over an average of 3.3 years follow-up, daily sedentary time increased by approximately 85 minutes, whilst LPA and MVPA decreased by approximately 77 and 8 minutes per day, respectively.

**Table 2:** Baseline, follow-up and change in sedentary time and physical activity intensities. Mean duration of follow-up 3.3 (1.6) years. Values are mean (SD) minutes per day, unless specified otherwise.

	Baseline		Follow-up		Change		% change†	
**Sedentary time**								
**All**	355.7	(76.1)	441.0	(82.2)	85.3	(80.6)	22.5	(33.7)
**Boys**	344.4	(73.7)	424.0	(85.0)	79.5	(80.7)	22.0	(33.7)
**Girls**	364.7	(76.8)	454.6	(77.2)	89.9	(80.2)	22.9	(33.8)
**LPA**								
**All**	388.5	(66.1)	311.4	(71.7)	−77.1	(70.2)	−19.4	(23.0)
**Boys**	388.8	(63.9)	318.7	(71.7)	−70.0	(68.8)	−17.5	(22.5)
**Girls**	388.3	(67.8)	305.5	(69.5)	−82.7	(70.8)	−20.8	(23.2)
**MVPA**								
**All**	55.9	(26.9)	47.7	(26.2)	−8.2	(32.1)	−16.2	(61.5)
**Boys**	66.8	(28.5)	57.4	(28.5)	−9.5	(32.1)	−15.3	(57.5)
**Girls**	47.0	(21.9)	39.9	(21.2)	−7.1	(24.7)	−17.1	(65.0)

All, n=6567; Boys n=2923; Girls n=3644.

†Median (inter-quartile range).

Data are presented for first and last points of assessment for each participant.

SD, standard deviation; LPA, light physical activity; MVPA, moderate to vigorous physical activity.

Annual change in sedentary time and physical activity intensity, stratified by day of the week, sex, age at baseline, BMI category, maternal education and ethnicity is presented in Table 3. Across the week and for all studied sub-groups, daily sedentary time increased by approximately 25 minutes/year, LPA decreased by approximately 22 minutes/year and daily MVPA decreased by approximately 3 minutes/year. Annual change estimates were statistically significant (all p < 0.001) for all outcomes.

**Table 3:** Annual change in sedentary time and physical activity intensities, stratified by day of the week, demographic and anthropometric characteristics.

	Sedentary time		LPA		MVPA	
	β	(95% CI)	β	(95% CI)	β	(95% CI)
**Day of the week (n = 6567)**						
**All days**	24.9	(24.5, 25.3)*	−22.4	(−22.8, −22.1)*	−2.5	(−2.6, −2.3)*
**Weekdays**	25.1	(24.7, 25.6)*	−22.8	(−23.2, −22.4)*	−2.3	(−2.5, −2.1)*
**Weekend**	24.4	(23.9, 25.0)*	−21.4	(−21.9, −20.9)*	−3.0	(−3.3, −2.8)*
**Sex**						
**Boys (n = 2923)**	23.6	(23.0, 24.2)*	−20.8	(−21.4, −20.3)*	−2.7	(−3.0, −2.5)*
**Girls (n = 3644)**	25.9	(25.4, 26.4)*	−23.6	(−24.1, −23.2)*	−2.2	(−2.4, −2.0)*
**Age at baseline**						
** < 10y (n = 1600)**	23.5	(22.9, 24.2)*	−20.9	(−21.5, −20.4)*	−2.6	(−2.8, −2.3)*
** > 10y (n = 4967)**	27.0	(26.5, 27.6)*	−24.6	(−25.1, −24.1)*	−2.4	(−2.6, −2.2)*
**Body mass index group**						
**Underweight (n = 549)**	26.0	(24.5, 27.4)*	−23.3	(−24.6, −22.0)*	−2.7	(−3.2, −2.1)*
**Normal (n = 4688)**	24.9	(24.5, 25.4)*	−22.3	(−22.7, −21.8)*	−2.6	(−2.8, −2.4)*
**Overweight/obese (n = 1321)**	24.4	(23.5, 25.3)*	−22.6	(−23.4, −21.8)*	−1.8	(−2.1, −1.4)*
**Maternal education**						
**Low-mid (n = 1565)**	24.1	(23.3, 24.9)*	−21.5	(−22.2 −20.8)*	−2.5	(−2.8, −2.2)*
**High (n = 4097)**	24.9	(24.4, 25.4)*	−22.6	(−23.0, −22.1)*	−2.3	(−2.5, −2.1)*
**Ethnicity**						
**White (n = 4748)**	24.6	(24.1, 25.1)*	−22.5	(−23.0, −22.1)*	−2.1	(−2.3, −1.9)*
**Non-white (n = 326)**	22.9	(20.8, 24.9)*	−21.4	(−23.3, −19.5)*	−1.4	(−2.2, −0.6)*

LPA, light physical activity; MVPA, moderate to vigorous physical activity.

*p < 0.001.

Table 4 shows results from analyses examining whether the annual change in sedentary time or physical activity varied by day of the week, sex, age, body mass index group, maternal education or ethnicity. The annual increase in sedentary time was greater in girls compared to boys and in adolescents compared to children. The annual decrease in LPA was greater in girls compared to boys, in adolescents compared to children and for weekdays compared to during the weekend. The annual decrease in MVPA was greater in boys compared to girls and at the weekend compared to during the week. Participants who were overweight or obese at baseline assessment showed smaller annual reductions in MVPA compared to children who were normal weight. In all instances, between-group differences in behaviour change with age were small in magnitude, such that none exceeded 5 minutes per day.

**Table 4:** Annual change in sedentary time and physical activity intensities; effect modification by day of the week, demographic and anthropometric characteristics.

	Sedentary time			LPA			MVPA		
	β	(95% CI)	P	β	(95% CI)	P	β	(95% CI)	P
**Day of the week**									
**Age**	25.0	(24.5, 25.5)	<0.001	−22.9	(−23.3, −22.5)	<0.001	−2.2	(−2.4, 2.1)	<0.001
**Day (Ref: weekdays)**	−31.9	(−38.4, −25.4)	<0.001	−19.5	(−25.1, −13.9)	<0.001	−0.7	(−3.5, 2.1)	0.615
Age*Day of week	−0.2	(−0.4, 0.7)	0.526	1.0	(0.6, 1.4)	<0.001	−0.8	(−1.1, 0.6)	<0.001
**Sex**									
**Age**	23.5	(22.9, 24.1)	<0.001	−20.7	(−21.2, −20.2)	<0.001	−2.8	(−3.0, −2.5)	<0.001
**Sex (Ref: Boys)**	−9.5	(−18.9, −0.1)	0.050	33.1	(25.0, 41.3)	<0.001	−23.7	(−27.4, −20.1)	<0.001
Age*Sex	2.5	(1.8, 3.3)	<0.001	−3.0	(−3.7, −2.4)	<0.001	0.5	(0.2, 0.8)	<0.001
**Age group**									
**Age**	23.4	(22.8, 24.0)	<0.001	−20.8	(−21.3, −20.3)	<0.001	−2.6	(−2.8, −2.3)	<0.001
**Age-group (Ref: < 10y)**	−59.1	(−69.8, −48.4)	<0.001	61.2	(52.0, 70.4)	<0.001	−2.3	(−6.5, 1.9)	0.277
Age*Age-group	3.5	(2.7, 4.3)	<0.001	−3.7	(−4.5, −3.0)	<0.001	0.2	(−0.1, 0.5)	0.240
**Body mass index group**									
**Age**	24.9	(24.4, 25.4)	<0.001	−22.3	(−22.7, −21.9)	<0.001	−2.6	(−2.8, −2.4)	<0.001
**Weight (Ref: normal)**	8.5	(−3.6, 20.7)	0.167	7.6	(−2.9, 18.1)	0.155	−15.6	(−20.3, −10.9)	<0.001
Age*Weight	−0.2	(−1.1, 0.8)	0.752	−0.7	(−1.5, 0.1)	0.084	0.8	(0.5, 1.2)	<0.001
**Maternal Education**									
**Age**	24.2	(23.5, 25.0)	<0.001	−21.8	(−22.4, −21.1)	<0.001	−2.4	(−2.7, −2.1)	<0.001
**Mat Ed (Ref: Low-mid)**	0.9	(−10.3, 12.0)	0.879	0.7	(−8.9, 10.4)	0.879	−1.4	(−5.6, 2.9)	0.524
Age*Mat Ed	0.7	(−0.2, 1.5)	0.136	−0.7	(−1.5, < 0.1)	0.061	<0.1	(−0.3, 0.4)	0.815
**Ethnicity**									
**Age**	24.5	(24.1, 25.1)	<0.001	−22.5	(−22.9, −22.1)	<0.001	−2.1	(−2.3, −1.9)	<0.001
**Ethnicity (Ref: White)**	19.3	(−8.7, 47.2)	0.177	−9.9	(−34.2, 14.5)	0.427	−8.7	(−19.6, 2.2)	0.117
Age*Ethnicity	−0.7	(−2.9, 1.5)	0.533	0.3	(−1.6, 2.2)	0.741	0.3	(−0.5, 1.2)	0.469

LPA, light physical activity; MVPA, moderate to vigorous physical activity. Example interpretation: The test for effect modification indicated that annual change in sedentary time was greater in girls than boys (P < 0.001). Sedentary time increased by 23.5 min/day/year in boys and 26.0 min/day/year in girls (23.5 + 2.5).

## Discussion

In this large, multi-country pooled analysis, we found consistent evidence for an age-related decline in LPA and MVPA and a concomitant increase in time spent sedentary. This overall pattern of findings was consistent across days of the week and in selected demographic subgroups. Small differences in the magnitude of change by sex and age at baseline may be used to inform the timing and targeting of behaviour change interventions.

### Comparison with previous research

The observed reduction in physical activity with age is consistent with findings from existing systematic reviews and pooled analyses. Farooq et al [27], for example, reported a mean annual reduction in MVPA of 3.4 minutes per day in their synthesis of 52 studies that assessed physical activity by accelerometry in children and adolescents. The decline in MVPA emerged at approximately age 6 years in girls and 9 years in boys; they also noted that it was greater for weekend days compared to during the week, consistent with our findings and emerging evidence that heath behaviour interventions may be best targeted at unstructured days, such as weekends and school holidays [28,29]. In their pooled, cross-sectional analysis of 30 European studies, Steene-Johannessen et al [5] reported a reduction of approximately 3 minutes per day in MVPA between adjacent age groups (1-y increments from 2-3y to 17y), comparable to the magnitude of change reported here. Interestingly, a recent review of self-reported, domain-specific physical activity noted varying patterns of change in active transport, organised and non-organised activity during childhood and adolescence [30]. Despite the known limitations of reported physical activity, these findings suggest that in the context of an overall decline in activity, the relative quantity and contribution of activity in particular domains may vary. These findings should be corroborated by analyses based on device-assessed activity, through advanced processing/analytical techniques or synthesis of data from multiple sensors (e.g., accelerometry and GPS) [31]. This point notwithstanding, knowledge of changes in domain specific activity is complimentary to the evidence on changes in overall- or intensity-specific activity, highlighting specific behaviours that can be targeted in intervention programmes.

Across the whole sample, the annual increase in sedentary time was approximately 24 min/day, with slighter larger increases observed in girls (compared to boys) and adolescents (compared to children). This is comparable to the approximately 28 min/day average increase over 1 year found in a recent systematic review of change in accelerometer assessed sedentary time [32]. This is also consistent with evidence that specific sedentary behaviours, including TV viewing, computer use and time spent studying (inc. doing homework) increase to varying degrees during childhood and adolescence [32,33]. As noted above, these behaviour-specific data are a valuable compliment to device-based data on overall sedentary time, providing more specific targets for behaviour change interventions. Further elaboration of how age-related changes in specific sedentary behaviours vary across sociodemographic subgroups will also be valuable in this regard. Programmes aimed at reducing sedentary behaviour in young people have had some success, but effects have typically been small and the evidence is heavily weighted towards limiting TV viewing and/or computer use [34,35]. An impediment to the development of sedentary behaviour interventions is that the epidemiological evidence often lags significantly behind contemporary behaviour patterns and preferences. This is important because emerging evidence indicates that screen and sedentary behaviours may have both beneficial and detrimental associations with health and development depending on behaviour type, content and context [36–38]. Taken together, the evidence supports public health action to limit the age-related decline in physical activity and promote health-enhancing screen/sedentary behaviour patterns, but a carefully pitched and specific approach to modifying these behaviours is required to maximise benefit [29,39].

Our analysis revealed an approximately 22 min/day annual decline in LPA across the whole sample, comparable to age-related reductions on LPA described in previous longitudinal studies [40,41]. The decline in LPA was greater in both absolute and relative terms than that observed for MVPA. Evidence of the health benefits of LPA has evolved rapidly in recent years, with some longitudinal studies demonstrating stronger associations with health markers for LPA compared to MVPA [14,15,42,43]. Given the extent of the decline in LPA observed in this study, further research to establish the relative health benefits of activity across the intensity spectrum is warranted. This is consistent with recommendations from the WHO 2020 Physical Activity and Sedentary Behaviour Guideline Development Group who identified the need for better evidence on the health benefits of LPA, including the impact of breaking up sedentary time with LPA [44]. In addition, given the relatively modest effectiveness of interventions to promote MVPA in young people to date [10,45], further development and evaluation of behaviour change interventions aimed at maintaining or increasing LPA may be appropriate and complimentary to efforts to promote MVPA [46].

In interaction analyses, the annual increase in sedentary time and reduction in LPA was greater in adolescents (≥10y) than children (<10y), but no difference was observed for MVPA. These findings are counter to the once widespread belief that the age-related decline in activity begins during adolescence. This view has also been refuted by others [47] and is corroborated by recent cross-sectional data indicating an approximately linear reduction in MVPA from mid-childhood through to mid- to late-teens [5]. Taken together, this evidence supports the delivery of programmes to increase or maintain levels of MVPA throughout childhood and adolescence. Our finding of a steeper increase in sedentary time during adolescence compared to childhood may be attributable to a concentration of factors that facilitate sitting in this older age-group, including changes in free-time activities (facilitated by increasing autonomy), changes in the school environment and time spent on school/homework. However, our findings are in contrast to review evidence which indicated no difference in the age-related change in accelerometer-assessed sedentary time between children and adolescents [32]. These contrasting findings may be attributable to methodological factors or secular changes in how sedentary time patterns evolve with age, which may hinder the assessment of developmental changes central to this analysis and limit cross-study comparisons.

We also examined whether age-related changes in behaviour varied by sex, ethnicity or maternal education. No differences were observed between ethnic groups or by maternal education groups, but girls exhibited larger age-related increases in sedentary time and larger reductions in LPA compared to boys, whilst boys showed larger reductions in MVPA. Such information may be informative for intervention targeting but should be considered alongside absolute levels of behaviour. For example, despite exhibiting a greater decline in MVPA with age, boys accumulated approximately 20 minutes/day more MVPA than girls at all assessments used in this analysis. Thus, low levels of activity at baseline may have limited scope for further reductions in MVPA amongst girls. In a similar vein, white and non-white participants exhibited similar patterns of change in behaviour over time, but non-white participants had consistently higher levels of sedentary time and lower MVPA. On this basis, girls and children of non-white ethnicity may benefit from targeted intervention programmes, but this should be subject to consultation with such groups (including parents) to establish the acceptability of this approach and appropriate intervention structure and content.

### Strengths and limitations

Strengths of this analysis include the large, international sample and device-based assessment of physical activity and sedentary time on at least two occasions. Accelerometer data were re-processed under a common protocol and previously validated count thresholds applied to define activity intensity. We report age-related changes in activity intensity across a range of demographic subgroups and week/weekend days, which is valuable for informing the targeting and timing of behaviour change interventions. We acknowledge the following limitations. Firstly, we focus only on behaviour change associated with chronological age, without consideration of biological age which may vary within children of similar chronological age and exert an independent influence on behaviour patterns [48]. Disentangling the influence of chronological age and maturation on the trajectory of physical activity and sedentary behaviour during childhood and adolescence would be a valuable avenue for future research. We used BMI-derived categories to indicate participants weight status due to the widespread availability of this data across included studies but acknowledge its limitations as a measure of adiposity. Drop out analysis indicated potential for selection bias in the analytical sample due to missingness in accelerometer or socio-demographic data. The sample, nonetheless, remained sufficiently heterogenous to allow for the intended effect modification analyses. In one study included in this pooled analysis (Project TAAG, n = 269, ~ 4% of the analytical sample), maternal education was child-reported, potentially resulting in a greater degree of misclassification than those studies in which maternal education was self-reported. To enable reprocessing across studies, accelerometer data were collapsed to 60 second epochs; this may have resulted in underestimation of MVPA in younger children where activity tends to be more sporadic. Accelerometer data were collected between 1997−2013 thus data may not be reflective of current behaviour patterns. The limited available evidence suggests there have been modest secular changes in physical activity and sedentary behaviour over time, but we are aware of no evidence indicating that within-person changes associated with ageing have also changed over time [4,49]. Lastly, all data pre-date the COVID-19 pandemic starting March 2020; current evidence indicates that physical activity and sedentary behaviour patterns were impacted by the pandemic but long-term effects on behaviour patterns and changes with age remain unknown at this time.

## Conclusion

To our knowledge, this is the largest, multi-country pooled analysis of accelerometer assessed change in physical activity and sedentary time in young people. For each additional year of age, children spent approximately 25 minutes per day more time sedentary and three minutes per day less time in MVPA. Given the known benefits of physical activity throughout the life-course, findings support the development and implementation of public health policies and interventions to maintain or increase activity during childhood.

## Supporting information

S1 Table Demographic characteristics of participants at baseline and follow-up duration. Values are mean (SD) unless specified otherwise.
(DOCX)
