# Supplementary material for: Age-related change in children’s physical activity and sedentary time: The International Children’s Accelerometry Database (ICAD)
Source: PLoS One. 2025 Sep 10;20(9):e0327394. doi: 10.1371/journal.pone.0327394 (PMC12422510; doi:10.1371/journal.pone.0327394)
Supplement: S1 Table — Values are mean (SD) unless specified otherwise. (DOCX) [file pone.0327394.s001.docx]

**Table S1. Demographic characteristics of participants at baseline and follow-up duration. Values are mean (SD) unless specified otherwise.**

| **Study** | **Sample size*** | | **Age** | | **BMI**  **z-score** | | **Ethnicity**  **% white** | **Maternal Education**  **% low/medium SEP** |  | **Duration of follow-up (years)**** | | | |
| --- | --- | --- | --- | --- | --- | --- | --- | --- | --- | --- | --- | --- | --- |
|  | **N** | **(% Male)** |  |  |  |  |  |  |  | **T_1_** | | **T_2_** | |
| **ALSPAC** | 3225 | (47.3) | 11.9 | (0.6) | 0.3 | (1.2) | 89.6 | 20.8 |  | 2.2 | (0.6) | 3.7 | (0.3) |
| **Ballabeina** | 74 | (56.8) | 5.1 | (0.6) | 0.2 | (0.8) | N/A | 31.1 |  | 0.8 | (<0.1) | - | - |
| **CLAN** | 450 | (46.4) | 9.7 | (2.7) | 0.6 | (1.0) | - | 49.2 |  | 3.1 | (0.6) | 4.9 | (0.2) |
| **CoSCIS** | 160 | (45.6) | 7.3 | (1.1) | 0.3 | (1.1) | - | - |  | 3.4 | (1.3) | 6.6 | (0.2) |
| **EYHS Denmark** | 356 | (39.9) | 9.7 | (0.4) | 0.3 | (1.0) | 94.4 | 16.9 |  | 5.9 | (0.3) | - | - |
| **EYHS Norway** | 52 | (48.1) | 9.7 | (0.3) | 0.3 | (1.0) | 90.4 | 30.8 |  | 6.2 | (0.2) | - | - |
| **EYHS Portugal** | 66 | (37.9) | 9.7 | (0.3) | 0.7 | (1.2) | 97.0 | 81.8 |  | 7.2 | (0.3) | - | - |
| **HEAPS** | 197 | (51.3) | 8.2 | (2.6) | 0.7 | (1.0) | - | 45.2 |  | 3.1 | (0.2) | - | - |
| **IBDS** | 535 | (49.4) | 6.9 | (2.1) | 0.6 | (1.2) | 94.4 | 20.5 |  | 3.0 | (1.3) | 5.6 | (1.5) |
| **KISS** | 104 | (51.9) | 8.9 | (2.2) | 0.2 | (1.1) | - | 13.5 |  | 0.7 | (0.1) | - | - |
| **PEACH** | 394 | (40.4) | 11.1 | (0.5) | 0.2 | (1.1) | 87.5 | 29.9 |  | 1.9 | (1.4) | 4.4 | (0.5) |
| **SPEEDY** | 685 | (44.2) | 10.3 | (0.4) | 0.4 | (1.1) | 95.5 | 39.1 |  | 1.5 | (1.1) | 4.1 | (0.1) |
| **Project TAAG** | 269 | N/A | 11.9 | (0.4) | 0.6 | (1.3) | 52.0 | 26.8 |  | 2.0 | (0.1) | - | - |

*Sample size denotes number of participants providing valid accelerometer data for at least 2 waves of assessment.

**Follow-up duration calculated as elapsed time in years from date of first day of accelerometer assessment at baseline (T_0_) to corresponding day at each subsequent wave of assessment.

SD, standard deviation; N/A, not applicable; ALSPAC, Avon Longitudinal Study of Parents and Children; CLAN, Children Living in Active Neighbourhoods; CoSCIS, Copenhagen School Child Intervention Study; EYHS, European Youth Heart Study; HEAPS, Healthy Eating and Play Study; IBDS, Iowa Bone Development Study; KISS, Kinder-Sportstudie; PEACH, Personal and Environmental Associations with Children's Health; SPEEDY, Sport, Physical activity and Eating behaviour: Environmental Determinants in Young people; Project TAAG, Trial of Activity for Adolescent Girls.
